# Suckling by *Schistosoma mansoni*-infected mothers restored IgG2a and TGF-β production, but not IL-6 and delayed-type hypersensitivity in IL-12/IL-23-deficient mice

**DOI:** 10.1590/0037-8682-0744-2020

**Published:** 2021-03-22

**Authors:** Fabiana Leticia da Silva, Maria da Conceição Silva, Gabriela Calixto Ribeiro de Holanda, Eridan de Medeiros Coutinho, Silvia Maria Lucena Montenegro, Clarice Neuenschwander Lins de Morais, Valdênia Maria Oliveira de Souza

**Affiliations:** 1 Fundação Oswaldo Cruz, Instituto Aggeu Magalhães, Laboratório de Imunopatologia e Biologia Molecular, Recife, PE, Brasil.; 2 Universidade Federal de Pernambuco, Laboratório de Imunopatologia Keizo Asami, Setor de Imunologia, Recife, PE, Brasil.; 3 Universidade Federal de Pernambuco, Departamento de Ciências Farmacêuticas, Recife, PE, Brasil.

**Keywords:** Schistosomiasis mansoni, Suckling, Hypersensitivity

## Abstract

**INTRODUCTION:**

Suckling by schistosomotic mice improves anti-ovalbumin (OA) antibody production, while delayed-type hypersensitivity (DTH) remains unaffected. This property of milk from schistosomotic mice was investigated in IL-12/IL-23-deficient mice (IL-12p40KO).

**METHODS:**

We compared anti-OA DTH, IgG2a and cytokines in wild-type and IL-12p40KO mice suckled by infected (SIM) or non-infected (CONTROL) mothers.

**RESULTS:**

SIM mice showed similar intensity and eosinophils in the DTH, which was abolished in IL-12p40KO and IL-12p40KO-SIM mice. In IL-12p40KO-SIM, IgG2a and TGF-β levels were higher, but IL-6 levels were lower.

**CONCLUSIONS:**

Milk from schistosomotic mothers may evoke IgG2a without eliciting DTH in IL-12/IL-23 deficiencies, by changing TGF-β/IL-6 levels.

Maternal schistosomotic infections may interfere with the establishment of postnatal immunity^1^. Considering the effects of breast milk, apart from gestation, adult offspring mice showed increased antibody levels to ovalbumin (OA) heterologous antigen and improved ability of B cells to act as antigen presentation cells^2^ ^,^ ^3^. However, the cell-mediated response, evaluated by *in vivo* hypersensitivity reactions, did not show any alteration in its intensity, and tissue cellularity was not investigated^2^. 

IL-12 and IL-23 share the subunit IL-12p40, as well as the common chain IL-12Rβ1 of cell receptors. They direct the production of antibodies and cell influx into tissues^4^. IL-12 triggers responses to Th1 cells with IFN-γ, opsonizing and complement activators, IgG antibodies, and mononuclear cell infiltrate^3^. IL-23 acts to expand Th17 lymphocytes and neutrophil influx and is required for humoral and cell immune memory^5^ ^,^ ^6^. Although *S. mansoni* infection is marked by Th2 responses, antigen fractions from eggs and worms can elicit Th1 and Th17 responses in the host, as occurs in infected mothers^7^.

Considering that the IL-12 family plays an important role in both production of antibodies and for cell-mediated responses, we investigated whether antibody production would be sustained in the absence of IL-12/IL-23 cytokines and whether there would be a cellular component of DTH in the adult offspring of mice receiving breast milk from schistosomotic mothers.

Four-week-old female C57BL/6 wild-type (WT) mice were infected subcutaneously with 30 *Schistosoma mansoni* cercariae, Belo Horizonte (BH) strain. Sixty days post-infection, infected and non-infected WT mice had their estruses synchronized^2^ and WT male mice were caged at a 1:1 ratio. The same procedure was carried out in non-infected deficient IL-12p40 (IL-12p40KO) female and male mice to obtain IL-12p40KO newborns. Immediately after birth, WT and IL-12p40KO newborn mice were housed in cages with interchanged mothers. Some mice from WT non-infected mothers were suckled by WT infected mothers (Suckled by Infected Mothers-SIM); other mice from WT non-infected mothers were suckled by their own mothers (CONTROL). Some mice from IL-12p40KO mothers were suckled by WT infected mothers (IL-12p40KO-SIM), while others were suckled by WT non-infected mothers (IL-12p40KO). Six weeks later, male offspring (n=5) from the four study groups were subcutaneously immunized with OA (100 μg of protein/animal, Sigma Chemical Co., St. Louis, MO, USA), emulsified in complete Freund’s adjuvant (CFA-Sigma), at the base of the tail (0.1 ml/animal). The other male offspring remained free of immunization.

After eight days, the mice were challenged in the hind footpad with 2% aggregated OA, and the DTH reaction was measured 24 h later. On day 9 after immunization, the mice were bled and plasma samples were titrated for IgG1 and IgG2a antibodies using ELISA in 96-well plates (Nunc MaxiSorp, Roskilde, Denmark) sensitized with OA (20µg/ml) and biotinylated goat anti-mouse IgG1 or IgG2a (Southern Biotechnology Associates plates, Inc., AL, USA). The results were expressed as the mean of the sample optical density (OD) in the dilution within the linear part of the titration curve for each isotype ± standard deviation (1:512 for IgG1 and 1:32 for IgG2a). Spleen cell suspensions were aseptically obtained and cultivated (humidified incubator CO_2_; at 37 °C; 5×10^6^ cells/mL) with OA (500 μg/mL) or culture medium alone for 48h. Supernatants were assayed for cytokine content using sandwich ELISA with commercial kits MIF00 IFN-γ, M1700 IL-17, M5000 IL-5, M6000B for IL-6, M1000 IL-10, and MB100B TGF-β1 (all from R&D Systems Inc., Minneapolis, MN, USA). The detection limits were 9.4 pg/ml for IFN-γ; 10.9 pg/ml for IL-17; 15.6 pg/mL for IL-5; 7.8 pg/ml for IL-6; 31.2 pg/ml for TGF-β; 15.6 pg/mL for IL-10. Histopathological analyses of footpads (hematoxylin-eosin stain, HE) from animals were compared using the following classification (mean of the analysis of three microscope slides per group): absent, no eosinophils or neutrophils; light, scarce eosinophils or neutrophils; moderate, 10 to 15 eosinophils or neutrophils; intense, 20 or more eosinophils or neutrophils. 

Statistical analyses were performed using one-way analysis of variance, followed by multiple comparisons using Tukey’s method by using GraphPad Prism 5.0 (GraphPad Software, San Diego, CA, USA), with the significance level set at 0.05. The results are representative of the three experiments. The animal protocol was approved by the Ethical Commission on Animal Use of the Aggeu Magalhães Institute, FIOCRUZ, Recife, Pernambuco, Brazil (01/2010) and was in accordance with the ethical principles in animal research adopted by the Brazilian College of Animal Experimentation. 

Anti-OA DTH was similar in both OA-immunized SIM and CONTROL groups; however, anti-OA IgG2a production was higher in the SIM group (Table 1). In IL-12p40-deficient mice, the anti-OA DTH reaction was abolished, while anti-OA IgG2a levels were significantly higher in the IL-12p40KO-SIM group than in the IL-12p40KO group. There were no significant differences in anti-OA IgG1 levels among the groups (data not shown).

**TABLE 1:** OA-specific *in vivo* delayed-type hypersensitivity, IgG2a and cytokine synthesis in wild-type or IL-12p40 deficient adult mice suckled by *S. mansoni*-infected or uninfected mothers.

Groups^a^	DTH^b^	IgG2a^c^	Cytokine (pg/mL)^d^					
			IFN-γ	IL-17	IL-5	IL-6	TGF-β	IL-10
**Control**	71±25	0.2±0.3	400±29.8	330±28.0	ND	65±6.0	ND	104±8.6
**SIM**	94±36	0.5±0.3*	600±10.4	159±8.7*	57±6.0	169±1.8*	55±2.9	144±11
**IL-12p40KO**	28±15*	0.1±0.01	ND	30±5.5*^#^	35±5.2	46±3.9^#^	ND	95±9.2
**IL-12p40KO SIM**	38±19^#^	0.4±0.3^δ^	ND	23±1.4*^#^	52±3.9^δ^	24±1.5^#δ^	53±3.5	93±4.9

^a^ Wild-type (CONTROL) and IL-12p40-deficient mice (IL-12p40KO) suckled by uninfected mothers and wild-type (SIM) or IL-12p40KO-SIM mice suckled by *S. mansoni*-infected mothers immunized with OA+CFA. ^b^Footpad thickness increases at 24 h in response to OA. The results are presented as mean ±standard deviation. The mice were challenged with aggregated OA in the footpad 8 days after the OA+CFA immunization. ^c^OA-specific IgG2a antibody levels 9 days after OA+CFA immunization Plasma titration was performed using ELISA. The results represent the mean OD ±standard deviation of five mice at a 1:32 dilution. ^d^Cytokine content in the culture supernatants was assayed using two-site sandwich ELISA, and the results represent the mean of duplicate cultures plus standard deviation. The detection limits were 9.4 pg/ml for IFN-γ; 10.9 pg/ml for IL-17; 15.6 pg/mL for IL-5; 7.8 pg/ml for IL-6; 31.2 pg/ml for TGF-β; 15.6 pg/mL for IL-10. Nine days after OA+CFA immunization spleen cells were stimulated with OA (500 mg/mL) for 48 h (5 × 10^6^ cells). **ND:** non-detected. *Ρ<0.05 compared with CONTROL; ^#^P<0.05 compared with SIM; ^δ^ P<0.05 compared with IL-12p40KO.

The synthesis of IFN-γ was detected only in the supernatants of SIM and CONTROL mice, and there was no significant detectable difference. Lower levels of IL-17 were seen in SIM, IL-12p40KO, and IL-12p40KO-SIM mice than in CONTROL mice. IL-5 was detected in the SIM group but not in the CONTROL group. IL-5 levels in the IL-12p40KO-SIM group were higher than those in the IL-12p40KO group. IL-6 production was higher in SIM mice than in CONTROL mice. IL-6 synthesis was lower in the IL-12p40KO-SIM group than in the IL-12p40KO and SIM groups. TGF-β was only produced in the SIM and IL-12p40KO-SIM groups. IL-10 production was similar among the CONTROL and experimental groups.

All histological sections showed macrophages and a few plasma cells. However, in the CONTROL group (Figure 1A), there was an intense acute inflammatory tissue reaction that occupied the deep dermis (circle) and hypodermis, and penetrated the underlying musculature, with abscess foci (squares) and exudate consisting predominantly of normal and degenerated neutrophils (pus cells) (continuous black arrow). However, in the SIM group (Figure 1B), an intense and widespread acute inflammatory infiltrate was detected, with pronounced edema in the dermis (dotted black arrow, quadrant I) and hypodermis with large abscess foci and moderate eosinophilic infiltration was seen (dotted black arrow, quadrant II) invading the underlying muscular layer. In IL-12p40KO animals (Figure 1C), there was intense and diffuse acute inflammatory infiltration (red dotted rectangle) in the deep dermis and hypodermis, with predominance of neutrophils, a moderate amount of eosinophils, and abscess foci. In the IL-12p40KO-SIM group (Figure 1D), there was a diffuse acute inflammatory infiltrate mainly in the dermis with predominance of neutrophils and a moderate amount of eosinophils. 

**FIGURE 1:**
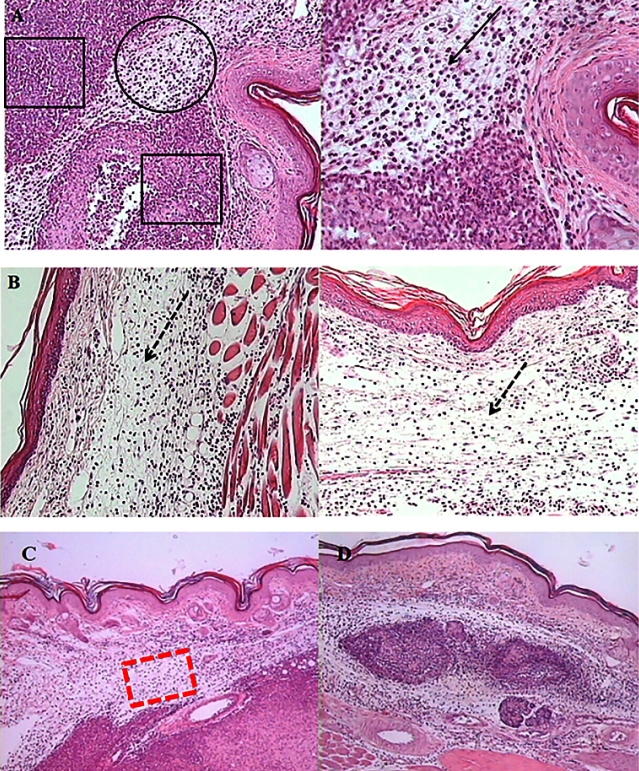
Cellular infiltrate in footpad skin of wild-type (SIM) or IL-12p40-deficient mice (IL-12p40-SIM) suckled by *S. mansoni*-infected mothers, as well as wild-type (CONTROL) and IL-12p40KO mice suckled by uninfected mothers, immunized with ovalbumin (OA) emulsified in complete Freund’s adjuvant (CFA) (OA+CFA). **(A)** CONTROL mice - deep dermis (circle), foci of abscess(squares), neutrophils and pus cells (continuous black arrow). **(B)** SIM mice - edema of the dermis (dotted black arrows), eosinophilic infiltration. **(C)** IL-12p40KO mice - diffuse acute inflammatory infiltrate (red dotted rectangle). **(D)** IL-12p40KO-SIM mice - diffuse acute inflammatory infiltrate. The mice were immunized with OA+CFA at the base of the tail and challenged intradermally in the footpads, with aggregated OA, 8 days after immunization (Hematoxylin-Eosin stain. **A**. 200x and 400x. **B**. 200x. **C** and **D**. 100x).

As expected, in the IL-12p40-deficient mice, there was no anti-OA IgG2a or IFN-γ production and lower levels of IL-17 were detected^4^ ^-^ ^6^. Nonetheless, anti-OA IgG2a levels were restored in IL-12p40KO-SIM mice, along with high production of IL-5 and TGF-β cytokines. Previous contact with breast milk from mothers infected with *S. mansoni* improved the antigen presentation ability of B lymphocytes for protein antigen^3^. IL-5 acts on B cells to induce proliferation and differentiation into antibody-secreting cells (ASCs) (plasma cell longevity)^8^. TGF-β favors the development of B lymphocytes and the production of IgA and IgG2b antibodies^5^. Therefore, these cytokines may contribute to IgG2a synthesis in the absence of IL-12/IL-23.

The SIM group showed features of a type 2 immune reaction (development of edema and eosinophils) in tissues in accordance with high levels of IL-5. In the inductor phase, the type 2 immune response modifies the morphology of cell-mediated response^9^. It is possible that early and continuous contact with the breast milk of infected mothers "primes" a bias toward Th2 response, favoring predominance of eosinophils even after immunization with OA emulsified in CFA. Therefore, this finding suggests that previous contact with breast milk from infected mothers may control disorders that are inherently Th1- and Th17-driven such as autoimmune diseases, which require a shift to Th2 response in early life^10^ ^,^ ^11^. 

However, the DTH in the IL-12p40-deficient mice was impaired in both the groups suckled by infected and non-infected mothers, as suggested by the diffuse and mixed cellular infiltrates seen in the footpads of these mice. The IL-12/IL-23 cytokine family is necessary for mononuclear and neutrophil influx^4^ ^,^ ^5^. IL-12 is required for eosinophil influx during the effector phase in the asthma model^12^. Interestingly, the absence of IL-12/IL-23 in association with previous contact with milk from schistosomotic mothers resulted in a dramatic reduction in the levels of IL-6, which modulates inflammatory processes, in contrast to the production of TGF-β, which is committed to immunosuppression. The low IL-6 levels in the present experiment can explain the minor degree of inflammation and abscess development in IL-12p40KO-SIM mice*.*

Taken together, these findings suggest that the dichotomous features (stimulatory and tolerogenic) of maternal milk^13^ were highlighted by *S. mansoni* infection in the absence of the IL-12 cytokine family. Breast milk from infected mothers has been previously shown to increase the expression of histone deacetylases (HDACs) involved in anti-inflammatory protein gene transcriptions^14^, while it contains proteins linked to the upgrading of glucose metabolism and the half-life of antigen-presenting cells, suggesting it promotes adaptive immunity as well as antibody production^15^. These results suggest that previous contact with breast milk may help in development of neutralizing antibody-dependent immunity in the immunodeficiency state linked to the Th1/Th17 profile (IL-12 family) response, as well as increase the suppressive environment by impairing IL-6 synthesis. The present study suggests that *S. mansoni* components could be used as immunomodulation tools in early life to provide a balance in humoral immunity and promote better control of inflammatory processes. These findings may support further research on pharmacological foods and nutraceuticals.
